# An easy, simple inexpensive test for the specific detection of *Pectobacterium carotovorum* subsp*. carotovorum* based on sequence analysis of the *pmrA* gene

**DOI:** 10.1186/1471-2180-13-176

**Published:** 2013-07-29

**Authors:** Mohamed Kettani-Halabi, Meriam Terta, Mohamed Amdan, El Mostafa El Fahime, François Bouteau, Moulay Mustapha Ennaji

**Affiliations:** 1 Laboratoire de Virologie, Microbiologie et Qualité /Eco Toxicologie et Biodiversité, Université Hassan II Mohammedia – Casablanca, Faculté des Sciences et Techniques - Mohammedia - FSTM, BP 146, Mohammedia 20650, Maroc; 2 UATRS – CNRST, Angle Allal Fassi / FAR, Hay Riad 10 000, Rabat, Maroc; 3 Université Paris Diderot, Sorbonne Paris Cité, LEM Institut de Biologie des Plantes, 91405 Orsay, France

**Keywords:** *pmrA* gene, *Pectobacterium carotovorum*, Potato tuber, Soft rot disease, Genetic diversity, Phylogenetic analysis

## Abstract

**Background:**

The species *Pectobacterium carotovorum* includes a diverse subspecies of bacteria that cause disease on a wide variety of plants. In Morocco, approximately 95% of the *P. carotovorum* isolates from potato plants with tuber soft rot are *P. carotovorum* subsp*. carotovorum.* However, identification of this pathogen is not always related to visual disease symptoms. This is especially true when different pathogen cause similar diseases on potato, citing as an example, *P. carotovorum*, *P. atrosepticum* and *P. wasabiae*. Numerous conventional methods were used to characterize *Pectobacterium* spp., including biochemical assays, specific PCR-based tests, and construction of phylogenetic trees by using gene sequences. In this study, an alternative method is presented using a gene linked to pathogenicity, in order to allow accuracy at subspecies level. The *pmrA* gene (response regulator) has been used for identification and analysis of the relationships among twenty nine *Pectobacterium carotovorum* subsp. *carotovorum* and other *Pectobacterium* subspecies.

**Results:**

Phylogenetic analyses of *pmrA* sequences compared to ERIC-PCR and 16S rDNA sequencing, demonstrated that there is considerable genetic diversity in *P. carotovorum* subsp. *carotovorum* strains, which can be divided into two distinct groups within the same clade.

**Conclusions:**

*pmrA* sequence analysis is likely to be a reliable tool to identify the subspecies *Pectobacterium carotovorum* subsp*. carotovorum* and estimate their genetic diversity.

## Background

*Pectobacterium carotovorum* subsp. *carotovorum* (*P. carotovorum* subsp*. carotovorum*) is a plant-pathogenic enterobacterium which belongs to the soft-rot group of *Pectobacterium*. It has the ability to cause serious damage worldwide on a numerous types of plants in field and storage stage [1]. In Morocco, approximately 95% of the *P. carotovorum* isolated from potato plants with tuber soft rot are *P. carotovorum* subsp*. carotovorum*[2]. This bacteria produce a wide variety of plant cell wall-degrading enzymes, causing maceration of different plant organs and tissues [1,3]. Many of its virulence genes have been identified, including genes encoding degradative enzymes, diverse regulatory systems, and the type III secretion system [4]. *Pectobacterium* spp*.* is a complex taxon consisting of strains with a range of different phenotype, biochemical, host range and genetic characteristics. Several methods were used to characterize this taxon, including biochemical assays and construction of phylogenetic trees by using gene sequences. For example, Toth and his collaborators [4-8] have shown that there are five major clades of *Pectobacterium* (formerly *E. carotovorum*): *atrosepticum*, *betavasculorum*, *carotovorum*, *odoriferum*, and *wasabiae*. Their analysis did not include *P. brasiliensis* which form individual clade [9]. Recently, other authors [10,11] were interested in molecular typing methods. These methods are increasingly used in the analysis of *P. carotovorum* subsp*. carotovorum* relatedness in order to identify their transmission routes and to assess its biodiversity. They have demonstrated a high diversity of polymorphism between these subspecies.

To survive, colonize and cause disease, plant-pathogenic bacteria modulate expression of their genes often using two-component signal transduction systems (TCS). These systems typically consist of two conserved components, a sensor histidine kinase and a response regulator [12]. *P. carotovorum* subsp. *carotovorum* employs different two-component systems for controlling production of virulence determinants [13-16]. PmrA-PmrB is one example of TCS for plant pathogenic bacteria, which affects production of extracellular enzymes, virulence and bacterial survival in potato tubers as well as in *Arabidopsis* leaves and generally *in planta*[17]*.* The main target genes of this TCS encode products with sequence similarity to DNA binding response regulators and autophosphorylatable histidine kinases.

The *pmrA* locus is required for resistance to the cationic peptide antibiotic polymyxin B and to other plant-derived antimicrobial peptides in *Pectobacterium*. It controls the production of proteins that mediate the modification of the lipopolysaccharide (LPS) core and lipid A [17-19]. The changes in LPS structure leads to reduction of the negative charges at cell surface and hence altered interactions with iron and cationic peptides [20]. This gene was found in almost all *Enterobacteriaceae*[20]. In *P. carotovorum* subsp*. carotovorum*, *pmrA* gene encodes a protein of 222 amino acid (aa) that reveals 59.7% of identity to *pmrA* of *Salmonella* and BasR of *E. coli*. Its inactivation in *P. carotovorum* subsp*. carotovorum* does not reduce the maceration ability of the bacterium on potato tuber but nevertheless remains essential for survival under adverse environmental conditions [16,20,21]. Phylogenies built with single genes have been used already to examine the relationships of the plant-pathogenic enterobacteria [22-25]. In this study, *pmrA* sequence analysis was used to identify the *Pectobacterium carotovorum* subsp*. carotovorum* and to estimate their genetic diversity. In addition, in at least one other system, this analysis was better correlated with Enterobacterial Repetitive Intergenic Consensus PCR (ERIC-PCR) assays and phylogenies built from 16S rDNA genes [10].

## Results and discussion

**Figure 1:**
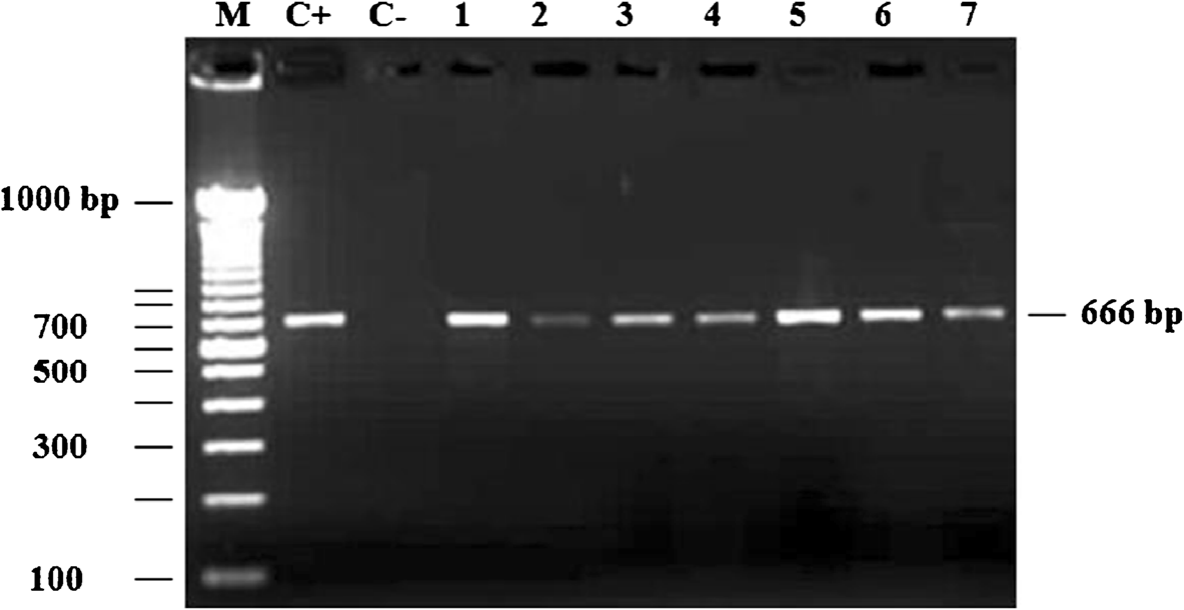
**Amplification of a specific 666 bp band in 7 strains generated using *pmrA *F0145 and E2477 primers.** Abbreviation: M, 100 bp DNA Step Ladder (1 kbp); C + (positive control), P116C2; C-, negative control 1, P111C2; 2, P111C3; 3, P111C4; 4, P211C1; 5, P211C2; 6, P211C3 and 7, P211C4.

**Figure 2:**
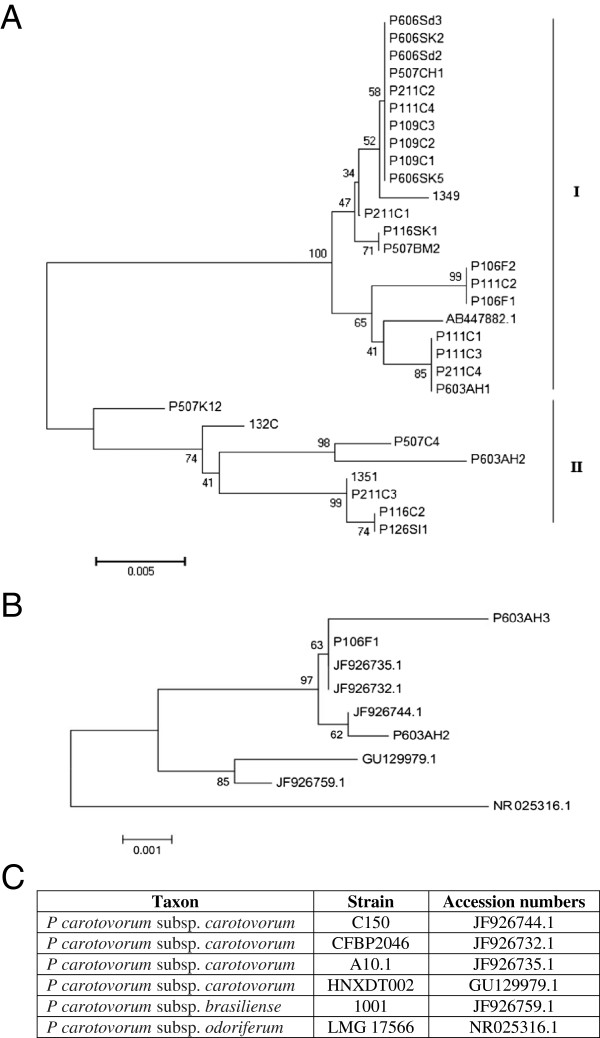
**Phylogenetic tree based on a comparison of *pmrA *sequences (A) and 16S rRNA (B) for *Pectobacterium carotovorum *subsp*. carotovorum. *(C)** Accession numbers of 16S rRNA sequences used for sequence alignments and construction of phylogenetic tree. The branching pattern was generated by the Neighbor-Joining method [31]. The numbers at the nodes indicate the levels of bootstrap support based on a Neighbor-Joining analysis of 500 resampled data sets. The evolutionary distances were computed using the Maximum Composite Likelihood method [32] and are in the units of the number of base substitutions per site. The generation of tree was conducted in MEGA5 [33].

**Figure 3:**
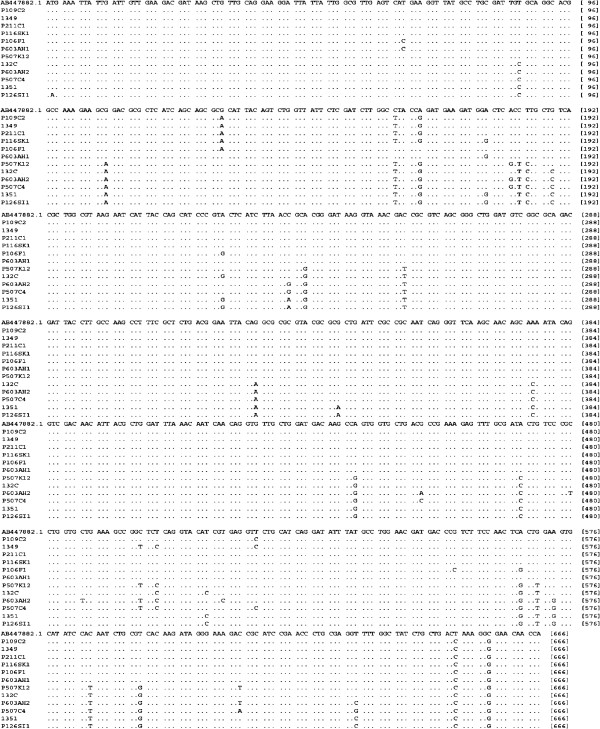
**Nucleic acid sequence alignment of *pmrA *gene among various strains of *Pectobacterium carotovorum *subsp. *carotovorum*.***P. carotovorum* subsp*. carotovorum pmrA* gene for response regulator *PmrA* (AB447882.1) available in GenBank was downloaded from NCBI. The alignments were performed using the ClustalW program [31]. The identical Nucleic acid in equivalent positions are indicated by dots and generated using the MEGA 5 program [32].

**Figure 4:**
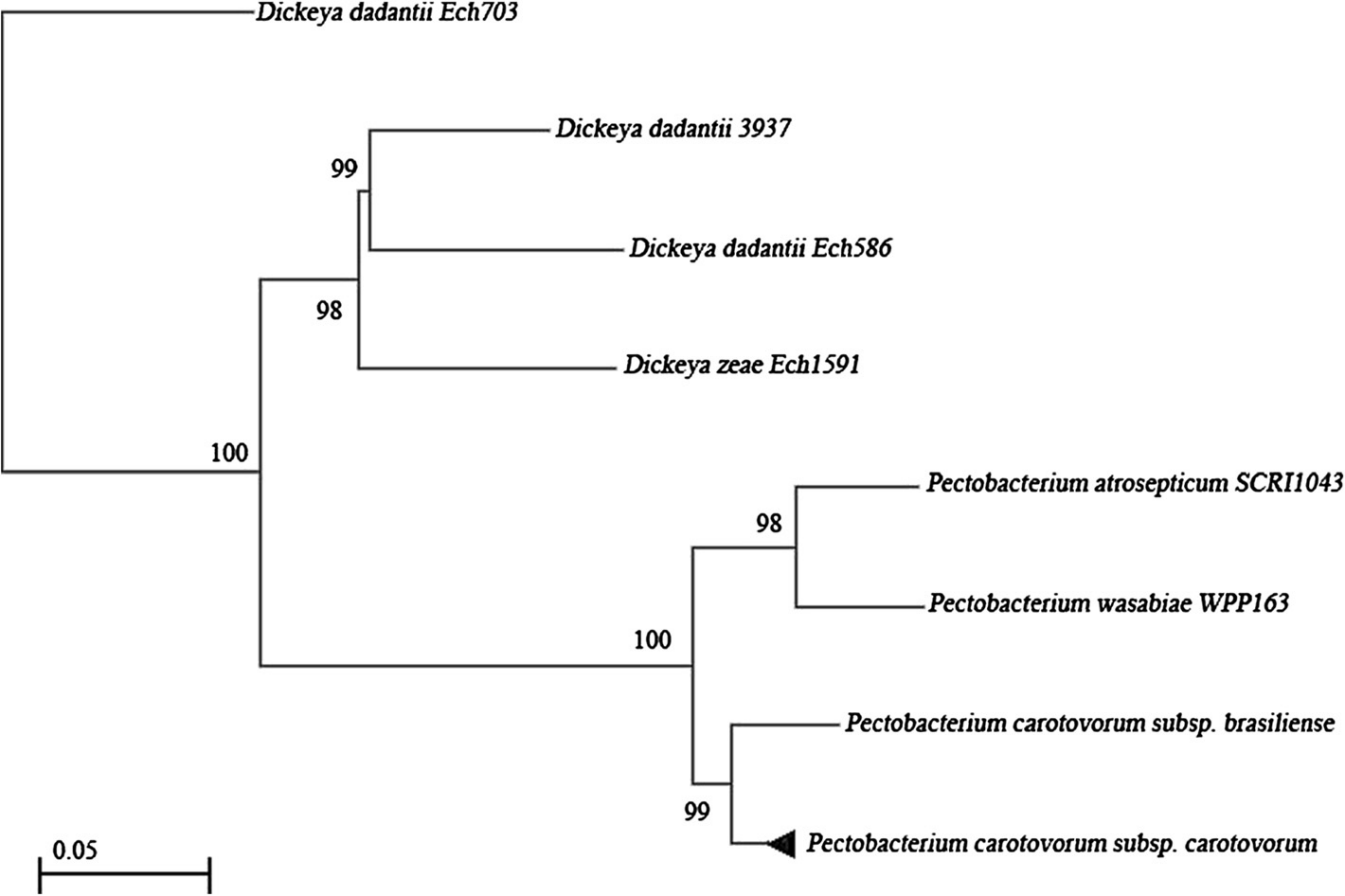
**Compressed subtree sequenced data for *pmrA *gene of 8 subspecies of *Enterobacteriaceae *based upon Neighbor-Joining method [**[33]**].** Subtrees presented in Figure 2 are compressed into black triangle. The numbers at the nodes indicate the levels of bootstrap support based on a Neighbor-Joining analysis of 500 resampled data sets. The evolutionary distances were computed using the Maximum Composite Likelihood method [34] and are in the units of the number of base substitutions per site. The generation of tree was conducted in MEGA5 [32].

Twenty-nine isolates from soft-rotted potato tubers (Table 1) were used in this study. They have been identified by biochemical and phenotypic tests ([2] and Additional file 1 Table S1). A part of the strains were already confirmed as *P. carotovorum* subsp*. carotovorum* using ERIC-PCR [2,10]. However, all strains yielded a 434 bp DNA fragment in PCR with the Y1 and Y2 specific primers for pectate lyase (pel) genes of *Pectobacterium* spp. [26,27] and a 666pb with specifics primers for *pmrA* of *Pectobacterium carotovorum* subsp. *carotovorum* (F0145 and E2477 [16]) (Figure 1). Our purpose in this study was to develop a tool with a high specificity to detect typical *Pectobacterium carotovorum* subsp. *carotovorum* isolated in Morocco and that could serve as a tool to evaluate the genetic diversity of these subspecies. To investigate the utility of *pmrA*-PCR as a method of identification, the dendrogram built (Figure 2A) from well-characterized strains was used to illustrate the clustering of subspecies, on the basis of a single-gene (*pmrA*) and analysis of 16 s rRNA gene sequences of *Pectobacterium* spp. (Figure 2B,C). Our phylogenetic tree (Figure 2A) revealed a high diversity among the subspecies tested with a maximum identity to the *pmrA* gene of strain WPP14 (AB447882.1), ranging from 95 to 99%. Moreover, phylogenetic distance between all strains is 0,02 suggesting that all *Pectobacterium carotovorum* subsp*. carotovorum* circulating in Morocco, have their origin from the United States [28,29]. Following numerical analysis of the 29 *pmrA* sequences by Neighbor-Joining (NJ) and UPGMA, the taxa were divided into two groups (clusters I to II), the similarity value between the two main clusters was about 96%. However, both clusters were represented by six different sequences (Figure 2A) and over 50% of the strains were included in the cluster I. Detailed scrutiny of the results given by the NJ method showed that all *P. carotovorum* subsp*. carotovorum* formed only one clade with 99% bootstrap. However, to verify the genetic diversity within our subspecies, the sequence alignment with maximum composite likelihood method (ML) were used. A comparison of 13 different *pmrA* sequences (Figure 3) revealed 0.05 as estimated value of the shape parameter for the discrete Gamma Distribution. The intraspecies comparison of DNA sequence identity is determined by the BLAST algorithm for *P. carotovorum* subsp*. carotovorum* strains for *pmrA* gene. This finding suggests that there is considerable genetic diversity in *P. carotovorum* subsp*. carotovorum* strains, which is in accordance with previous works reported by different authors [9,10,23,28]. Also, the multiple sequence alignment of these sequences revealed conserved regions at different stretches. These regions could be used for designing degenerate primers or probes for PCR-based amplification or hybridization-based detection of *pmrA* sequences from different subspecies of *P. carotovorum*. Furthermore, within the genus *Pectobacterium*, there are five major clades forming a polyphyletic group: *P. atrosepticum*, *P. betavasculorum*, *P. carotovorum* subsp. *carotovorum*, *P. odoriferum*[23], and *P. wasabiae*. These analyses did not include strains (*P. brasiliensis*[27]). Our phylogeny (Figure 4) places all the strains previously identified using biochemical and phenotypic methods in the group *P. carotovorum* subsp*. carotovorum*, noting that, some potato strains collected in different years and in widely different locations were grouped closely in the same group. It places also *P. brasiliensis* more similar to *P. carotovorum* subsp*. carotovorum* than to *P. atrosepticum* (*E. carotovora* subsp*. atroseptica* SCRI1043) and *P. wasabiae* WPP163, knowing that the level of similarity between the two *pmrA* sequences subspecies *P. atrosepticum* and *P. carotovorum* subsp. *carotovorum* is 98.19%. Many others phylogenetic analysis revealed that not all subspecies of *P. carotovorum* were grouped in a single, robust clade identified by all methods [9,29]. This was a strong indication that the different subspecies of *P. carotovorum* could indeed belong to different species. Despite the fact that some authors have concluded that the phylogenies built with single genes do not have many informative characters, and they “may not accurately reflect interspecies taxonomic relatedness” [22], our current phylogenetic analysis of *pmrA* sequences was clearly sufficient to determine whether all of these subspecies can be placed in the same subspecies or to split into two different subspecies. Noting that, the *pmrA* gene sequences have several advantages, including being effectively a single-copy gene, highly conserved in *P. carotovorum* subsp*. carotovorum* and easy to amplify. Therefore, the sequencing and analysis sequence data for the *pmrA* region of *P. carotovorum* subsp*. carotovorum* strains could be a reliable tool for detection of pathogens. Moreover, *pmrA* sequence analysis has shown a high genetic diversity among the isolates *P. carotovorum* subsp*. carotovorum.* The same results have been reported by other studies [2,5,9,23,29] using several phylogenetic analyses seeking to understand the relationship among these nominal subspecies.

**Table 1:** Strains used in this study

**Species/subspecies**^**a**^	**Accession no**	**Isolates**	**Year isolated**	**Moroccan city**	**Reference**
*P*. *carotovorum* subsp. *carotovorum*	JQ278721	P603AH1	2003	Ain halouf	[2,10]
	JQ278727	P106F1	2006	Fes	[2,10]
	JQ278728	P116SK1	2006	Sidi kacem	[2,10]
	JQ278731	P606SK2	2006	Sidi kacem	[2,10]
	JQ278738	P606SK5	2006	Sidi kacem	[2]
	JQ278736	P606Sd2	2006	Sidi slimane	[2,10]
	JQ278748	P126SI1	2006	Sidi issa	[2]
	JQ278749	P116C2	2006	Casablanca	[2,10]
	JQ278739	P507CH1	2007	Chtouka	[2]
	JQ278742	P507K12	2007	Kenitra	[2]
	JQ278724	P111C1	2011	Casablanca	This study
	JQ278744	P603AH2	2003	Ain halouf	[10]
	JQ278741	1349	2003	Ain halouf	[30]
	JQ278725	P106F2	2006	Fes	This study
	JQ278732	P606Sd3	2006	Sidi slimane	This study
	JQ278746	1351	2006	Casablanca	[30]
	JQ278743	P507C4	2007	Casablanca	This study
	JQ278729	P507BM2	2007	Beni mellal	[10]
	JQ278726	P111C2	2011	Casablanca	This study
	JQ278723	P111C3	2011	Casablanca	This study
	JQ278737	P111C4	2011	Casablanca	This study
	JQ278734	P109C1	2009	Casablanca	This study
	JQ278733	P109C2	2009	Casablanca	This study
	JQ278740	P109C3	2009	Casablanca	This study
	JQ278730	P211C1	2011	Casablanca	This study
	JQ278735	P211C2	2011	Casablanca	This study
	JQ278747	P211C3	2011	Casablanca	This study
	JQ278722	P211C4	2011	Casablanca	This study
	JQ278745	132C	2006	Casablanca	[30]

^a^ All strains have for hosts: potato and for *pmrA*-PCR product: 666 pb.

## Conclusions

Our *pmrA* gene sequence analysis, linked to pathogenicity studies, could be used to identify and monitor the diversity of the *P. carotovorum* subsp*. carotovorum* subspecies.

## Methods

### Sample handling and isolate bacteria

During the years 2003 to 2011, different potato fields and the most important potato storages were controlled in Morocco and several samples were collected from plants with soft rot disease. Nutrient agar, King’s B agar, Crystal Violet Pectate (CVP) and LPGA medium (5 g/L yeast extract, 5 g/L peptone, 5 g/L glucose 15 g/L agar) were used to isolate the suspected bacteria. The 29 strains used in this study are isolated from different geographic Moroccan regions and had been stored in 20% glycerol at −20°C [2,30]. Table 1 shows the strains whose sequences were determined in this study and the reference strains used for comparison when phylogenetic trees were constructed. Table 1 includes the strain designations and the GenBank accession numbers for the *pmrA* sequences.

### Biochemical and physiological tests

In order to identify *Pectobacterium* spp., the strains were grown at 27°C for 24 h on agar plates and they were tested for Gram staining, catalase, oxidase, nitrate production, reductase activity, pectinolytic activity on Sutton medium, and absence pigmentation of the strains in the King B medium (Difco) [2]. Identification of confirmed *Pectobacterium* spp*.* isolates to species and subspecies was conducted on the basis of biochemical tests (indole production from tryptophan, lecithinase activity and acid production from α-methyl glucoside, trehalose, sorbitol, melibiose, lactose). All tests were carried out at 27°C for 24 h and compared with the standard strains (see Additional file 1 Table S1 for the fourteen strains used only in this study) [2,10].

### DNA extraction and *PCR amplification*

Bacterial cultures from frozen stocks were grown onto LPGA medium and suspended in sterile H_2_O. The concentration was adjusted to 10^8^ CFU.ml^-1^. DNA was extracted from bacterial suspension as described by Terta et al. [2]. The precipitated DNA then was quantified using a NanoDrop 8000 spectrophotometer (Thermo Scientific, Wilmington, DE, USA), adjusted to 100 ng.μl^-1^ and stored at 4°C. All PCR amplifications were performed using the following primers: *pmr A* F0145 (5’-TACCCTGCAGATGAAATTATTGATTGTTGAAGAC-3’) and E2477 (5’-TACCAAGCTTTGGTTGTTCCCCTTTGGTCA-3’) as described by Hyytiäinen et al. 2003 [16]. A 25 μl PCR mix contained: 1 μl DNA, 0.5 U Taq DNA polymerase, 2.5 μl 10 × PCR buffer, 2.5 mM each of dNTPs, 2.5 mM MgCl2, 0.5 μM of each primer. DNA amplification was performed on Veriti® Thermal Cycler (Applied Biosystems) under the following conditions: 5 min at 94°C for initial denaturation, 35 cycles of 1 min at 94°C for, 1 min at 55°C and 2 min 72°C, followed by a final elongation step of 10 min at 72°C. PCR products (6 μl) were separated by gel electrophoresis in 1.8% agarose gels in TBE buffer. Following staining with ethidium bromide, the gels were viewed and photographed under UV Transilluminator. Fragment sizes were determined by comparison to a 100 bp DNA Ladders.

### Sequencing of *pmrA* and phylogenetic analysis

The PCR-amplified fragments of *pmrA* were purified and the sequencing reactions were performed with a Big-Dye Terminator v3.1 (Applied Biosystems). The *pmrA* sequences which we determined and the sequences of the reference strains of members of the family *Enterobacteriaceae* obtained from the GenBank databases were analyzed. The *pmrA* sequences were first aligned by using the Clustal W program [34], and then the alignments were corrected by hand. Evolutionary trees for the data set were inferred by using the Neighbor-Joining program of MEGA [31,33]. The stability of relationships was assessed by performing bootstrap analyses of the Neighbor-Joining data based on 500 resamplings. The entire sequences corresponding to positions 4317866-4318532 of the reference sequence of the subspecies.

### Nucleotide sequence accession numbers

The *pmrA* sequences which we determined have been deposited in the GenBank database under the accession numbers shown in Table 1.

## Competing interests

The authors declare that they have no competing interests.

## Authors’ contributions

MK-H designed the study, performed the experiments, data analyses and wrote the paper, MT and MA participated in the sample preparation and preliminary examination, EE participated in the design of the study, FB drafted the manuscript, MME coordinated the study, designed and participated in manuscript preparation. All authors read and approved the manuscript.

## Supplementary Material

Additional file 1: Table S1 Phenotypic characteristics of the strains of *Pectobacterium* isolated from potato in comparison with standard isolate. Click here for file
