# Supplementary material for: An easy, simple inexpensive test for the specific detection of Pectobacterium carotovorum subsp. carotovorum based on sequence analysis of the pmrA gene
Source: BMC Microbiol. 2013 Jul 29;13:176. doi: 10.1186/1471-2180-13-176 (PMC3765535; doi:10.1186/1471-2180-13-176)
Supplement: Additional file 1: Table S1 — Phenotypic characteristics of the strains of Pectobacterium isolated from potato in comparison with standard isolate. [file 1471-2180-13-176-S1.docx]

**Supplementary table 1.** Phenotypic characteristics of the strains of *Pectobacterium* isolated from potato in comparison with standard isolate.

|  | **Results** | |  |
| --- | --- | --- | --- |
| **Test** | **Number of strains positive or** | **Positive** | **Standard isolate** |
|  | **negative/number of strains tested** | **strains (%)** | ***P. carotovorum*** |
| Gram reaction | - (14/14) | 0 | - |
| Fermentative growth | + (14/14) | 100 | + |
| Potato soft rot | + (14/14) | 100 | + |
| Oxidase | - (14/14) | 0 | - |
| Catalase | + (14/14) | 100 | + |
| Lecithinase | - (14/14) | 0 | - |
| Gelatin hydrolysis | + (12/14) | 86.7 | + |
| Gas from glucose | - (13/14) | 6.6 | - |
| Growth at 37ºC | + (14/14) | 100 | + |
| Production of Indole | - (13/14) | 6.6 | - |
| Pigment production | - (14/14) | 0 | - |
| Nitrates reduction | + (14/14) | 100 | + |
| Pectinolytic activity | + (14/14) | 100 | + |
|  | Acid production from: |  |  |
| Glucose | + (14/14) | 100 | + |
| Fructose | + (14/14) | 100 | + |
| α-methyl-D-glucoside | - (14/14) | 0 | - |
| Sorbitol | + (12/14) | 86.7 | + |
| Melibiose | + (14/14) | 100 | + |
| Arabitol | - (14/14) | 0 | - |
| Lactose | + (14/14) | 100 | + |
| Trehalose | + (14/14) | 100 | + |

+, positive reaction; -, negative reaction.

The 14 strains correspond to the strains used only in this study (table 1).
